# A young non-immunocompromised woman with diffuse alveolar opacities

**DOI:** 10.4103/0970-2113.71959

**Published:** 2010

**Authors:** Prem Parkash Gupta, Manish Verma, Dipti Agarwal, Sanjay Kumar, Atulya Atreja

**Affiliations:** Department of Respiratory Medicine, Postgraduate Institute of Medical Sciences, University of Health Sciences, Rohtak, India; 1 Department of Physiology, Postgraduate Institute of Medical Sciences, University of Health Sciences, Rohtak, India; 2 Department of Pathology, Postgraduate Institute of Medical Sciences, University of Health Sciences, Rohtak, India

**Keywords:** Alveolar opacities, CT guided fine needle aspiration cytology, fiberoptic bronchoscopy, pulmonary tuberculosis

## Abstract

Diffuse alveolar opacities (DAO) due to pulmonary tuberculosis are usually described in immunocompromised patients. In adult patients residing in high endemic areas such as India, alveolar opacities are not reported frequently in non-immunocompromised pulmonary tuberculosis patients. We describe a twenty-five-year-old woman who presented with bilateral diffuse alveolar opacities and initial diagnostic work up was directed to non-tuberculosis etiologies. Her sputum was not suggestive of tuberculous or any other infective etiology. However, histopathological examination of specimen from fine needle aspiration cytology through percutaneous route suggested chronic granulomatous disease with detection of mycobacterium. Polymerase chain reaction test in BAL and FNAC specimen confirmed tubercular etiology. Though not frequent, pulmonary tuberculous etiology is worth considering in the differential diagnosis of DAO as not only tuberculosis is fully treatable but also early detection shall help to avoid unnecessary invasive tests and cut down transmission to contacts.

## INTRODUCTION

Alveolar opacities often present a diagnostic enigma to radiologists and pulmonologists alike. The classical alveolar opacities are characterized by (1) fluffy and ill-defined margins except where they abut upon a pleural surface, (2) coalescence of individual lesions with the adjacent one as the intervening alveoli become involved, (3) butterfly or bat’s wing distribution, and (4) presence of air-bronchogram manifested when the air filled bronchi are surrounded by infiltrates in alveoli.[1] When the alveolar signs are clear-cut, they do have a high degree of reliability. Though several of these classical appearances may be evident in an individual patient, even one of them is a sufficient sign of alveolar involvement. Diffuse alveolar involvement is observed in a wide variety of unrelated diseases; tuberculosis being responsible not frequently, and that too in immunocompromised subjects. WHO estimates that 9.27 million new cases of tuberculosis occurred worldwide in 2007 compared with 9.24 million new cases in 2006.[2] India ranks first in terms of the total number of incident tubercular cases.[2] Still, in this part of world where the person might have been immunized with BCG or might have had exposure to tubercular bacilli since childhood due to high prevalence in the community, it is not frequent to encounter diffuse alveolar opacities due to tuberculous etiology in non-immunocompromised adult host.

## CASE REPORT

**Figure 1:**
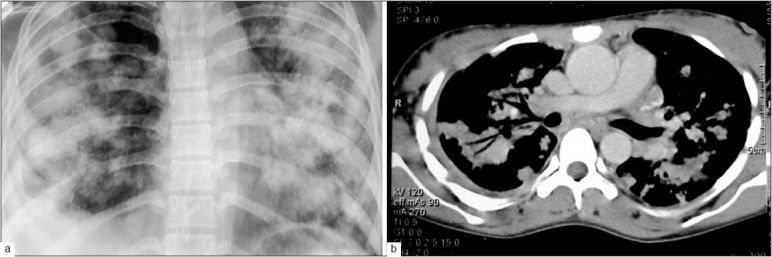
a) Chest radiograph PA view showing bilateral diffuse alveolar opacities having irregular fluffy margins, showing a pattern of coalescence and presentation with butterfly distribution. The blunting of right CP angle is suggestive of pleural effusion; b) CT thorax showing the diffuse alveolar opacities and presence of right pleural effusion

**Figure 2:**
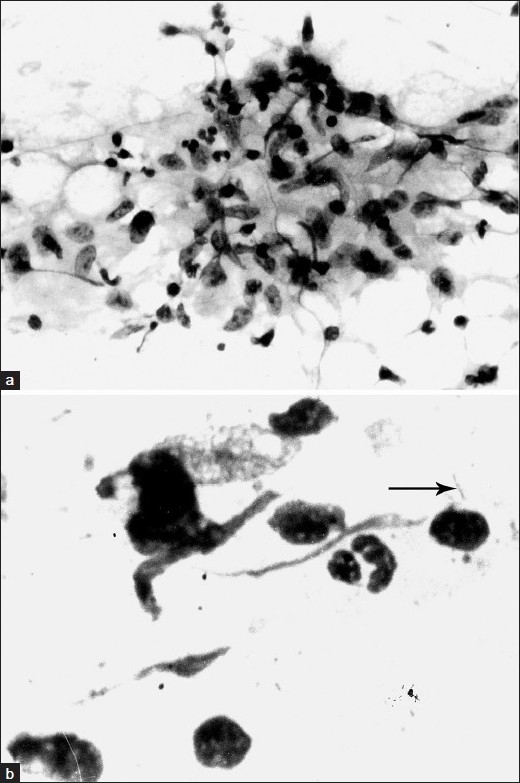
a) CT-guided FNAC smear showing epitheloid cells granuloma (MGG stain; 400X); b) CT-guided FNAC smear showing acid-fast bacillus, marked with arrow (ZN stain; 1000X)

A twenty-five-year-old woman presented at our Institute with three months history of cough with scanty expectoration, progressive dyspnoea and chest pain. She migrated from Nepal to India along with her husband and was working as a part-time domestic helper. There was no history of diabetes, hypertension, or any other significant disease. She had weight loss by 4 kg over the last three months, presently weighing 40 kg. She had dyspnoea at rest, respiratory rate 28/min, and pulse rate 120/min. She had wide spread crackles over bilateral lung fields. No significant cardiovascular system or ECG finding was observed. No cause of chronic alveolar edema could be elicited. There was no clinical evidence of any gynecological involvement. No BCG scar was seen and she could not recall any BCG vaccination. Her complete blood count, routine biochemical tests including blood sugar and urinalysis were within normal limits. Her serum was non-reactive for HIV-antigens on two different occasions. Chest radiograph revealed bilateral diffuse alveolar opacities having irregular fluffy margins with a pattern of coalescence and presenting in butterfly distribution. The blunting of right CP angle was also seen [Figure 1a]. The tuberculin test read at 48 h showed an induration of 28 mm. The microbiological evaluation of her sputum showed no pathogen. CT scan of thorax [Figure 1b] was also suggestive of alveolar opacities, showing the *air-bronchogram* within alveolar opacities and presence of right pleural effusion. Thoracentesis and the evaluation of pleural fluid showed exudative effusion but leading to no further differentiation. The various differential diagnoses considered [Box-1] were individually evaluated for by using fiberoptic bronchoscopy, detailed systemic history, thorough clinical examination and appropriate relevant investigations. The BAL specimen and brush specimen obtained through the fiberoptic bronchoscope were negative for any malignant cells and bacterial, mycobacterial or fungal infection using conventional methods. CT-guided fine needle aspiration cytology of the pulmonary lesions was carried out and that revealed epitheloid cells granuloma [Figure 2a] and the Z N stain for acid-fast bacilli was positive [Figure 2b] confirming the tuberculous lesion. Polymerase chain reaction (PCR) investigation in specimen from FNAC of lung lesions and BAL sample confirmed tubercular etiology. She was diagnosed to have pulmonary tuberculosis and was given WHO Category-I anti-tubercular treatment (2H_3_R_3_Z_3_E_3_/4H_3_R_3_). She had a favorable response with anti-tubercular chemotherapy.

**Box 1:** Differential diagnoses considered in present patient presenting with diffuse alveolar opacities

Sarcoidosis Alveolar cell carcinoma Alveolar form of lymphoma Alveolar proteinosis Fungal infections Choriocarcinoma, metastatic Tuberculosis

## DISCUSSION

As mentioned earlier, alveolar pattern may be encountered in a wide variety of unrelated diseases.[3,4] The word “diffuse” implies involvement of all lobes of both lungs, but the process need not affect all lobes or all lung regions uniformly. For differential diagnosis purpose, these diseases may be classified as acute or chronic conditions. The *acute alveolar pattern* is usually seen in pulmonary edema, pneumonia, hyaline membrane disease, aspiration pneumonia, pulmonary hemorrhagic disorders, etc. *Chronic pattern* may be seen in sarcoidosis, alveolar proteinosis, lymphomas, alveolar cell carcinoma, desquamative pneumonitis, mineral oil aspiration, alveolar microlithiasis, and also in chronic infections such as tuberculosis or fungal infections. The radiological manifestations of alveolar cell carcinoma are highly variable and are often indistinguishable from other lesions with similar appearance. The alveolar form of lymphoma may present as primary lesion of the lung or as a part of disseminated disease.[1,3] The persistent alveolar infiltrates have also been reported in cryptogenic organizing pneumonia[4] and chronic eosinophilic pneumonia.[5] Alveolar proteinosis usually present with typical radiological pattern of DAO.[6] In sarcoidosis the radiological appearance is due to encroachment on and obliteration of airspaces by an interstitial process and not due to diffuse involvement confined to alveoli.[7] In tuberculosis and fungal infection, DAO are usually described in immunocompromised patients such as those infected with HIV virus, during the course of lymphoma or during immunosuppressive therapy after organ transplantation.[8–10]

In the present case report, the patient had symptoms for three months and her lung opacities did not respond to antibiotic therapy administered by the general physician before she was referred to our institute. There were certain points during initial work-up that not favoring the diagnosis of the tuberculosis: the patient had no fever and had no known immunocompromised state. It has been documented that approximately 10% of patients who are subsequently proved to have tuberculosis had a pulmonary infiltrate that was thought not to be the characteristic of tuberculosis on radiographs.[8]

The CT-guided transthoracic needle aspiration of the pulmonary lesions suggested the tuberculous etiology that was supported by confirmation using PCR test. The presence of exudative pleural effusion with lymhocytic predominance was compatible with the diagnosis of tuberculosis. The present patient may serve a message that tuberculous etiology should be kept in the differential diagnosis of diffuse alveolar opacities even in high endemic regions and should be ruled out at an early stage as tuberculosis is fully treatable and the initiation of early antitubercular chemotherapy shall interrupt the chain of transmission to the contact persons and children. If missed during initial work-up; endless time-consuming, costly and invasive procedures shall be undertaken without leading to fruitful outcome and that will elevate the patient’s apprehension and decrease the faith in diagnostic methodology.

## References

[1] Felson B, Felson B (1988). The pulmonary airways. Chest roentgenology.

[2] WHO. Tuberculosis – Epidemiology. TB incidence, prevalence and mortality. http://www.who.int/tb/publications/global_report/2009/pdf/chapter1.pdf.

[3] Ryu JH, Olson EJ, Midthun DE, Swensen SJ (2002). Diagnostic approach to the patient with diffuse lung disease. Mayo Clin Proc.

[4] Vasu TS, Cavallazzi R, Hirani A, Sharma D, Weibel SB, Kane GC (2009). Clinical and radiologic distinctions between secondary bronchiolitis obliterans organizing pneumonia and cryptogenic organizing pneumonia. Respir Care.

[5] Jeong YJ, Kim KI, Seo IJ, Lee CH, Lee KN, Kim KN (2007). Eosinophilic lung diseases: A clinical, radiologic, and pathologic overview. Radiographics.

[6] Haga T, Kasamatsu N, Kobayashi T, Shibata M, Ogasawara T, Hashizume I (2009). A case of pulmonary alveolar proteinosis presenting with peripheral ground-glass opacitiy. Nihon Kokyuki Gakkai Zasshi.

[7] Park HJ, Jung JI, Chung MH, Song SW, Kim HL, Baik JH (2009). Typical and atypical manifestations of intrathoracic sarcoidosis. Korean J Radiol.

[8] Woodring JH, Vandiviere HM, Fried AM, Dillom ML, Williams TD, Melvin IG (1986). Update: The radiographic features of pulmonary tuberculosis. AJR Am J Roentgenol.

[9] Washington L, Miller WT (1998). Mycobacterial infection in immunocompromised patients. J Thorac Imaging.

[10] Wu JY, Ku SC, Shu CC, Fan JY, Chen HY, Chen YC (2009). The role of chest radiography in the suspicion for and diagnosis of pulmonary tuberculosis in intensive care units. Int J Tuberc Lung Dis.

